# A Generalized Convolutional Neural Network Model Trained on Simulated Data for Fault Diagnosis in a Wide Range of Bearing Designs

**DOI:** 10.3390/s25082378

**Published:** 2025-04-09

**Authors:** Amirmasoud Kiakojouri, Ling Wang

**Affiliations:** National Centre for Advanced Tribology at Southampton (nCATS), School of Engineering, University of Southampton, Southampton SO17 1BJ, UK; amirmasoud.kiakojouri@soton.ac.uk

**Keywords:** bearing fault diagnosis, data scarcity, generalisability, simulated vibration data, convolutional neural network (CNN)

## Abstract

Rolling element bearings (REBs) are critical components in rotating machinery and a leading cause of machine failures. Traditional fault detection methods rely on signal processing, but advances in machine learning (ML) and deep learning (DL) have dramatically improved diagnostic accuracy. However, existing DL models struggle with data availability, generalization, and domain adaptation, making industrial applications challenging. This study proposes a convolutional neural network (CNN) model trained on numerically simulated vibration data generated for a wide range of bearing designs. A novel hybrid signal processing method is employed to enhance feature extraction and reduce domain shifts between simulated and real-world data. The optimized CNN model, trained on simulated data, is tested using experimental and real-world vibration signals from laboratory bearings and jet engine components. The results show high classification accuracy using data from the Case Western Reserve University experimental dataset and successful fault detection in real-world Safran jet engine ground tests. The findings demonstrate the effectiveness of the developed CNN-based model for bearing fault classification, tackling training data scarcity and generalizability challenges while contributing to the development of intelligent fault diagnosis models for several industrial applications.

## 1. Introduction

Rolling element bearings (REBs), as one of the most prevalent components in rotating machines, are also the predominant causes of machine failures [1]. For example, 30–40% of all failures in induction machines [2,3] and about 7% in gas turbines [4] are caused by bearing failures. Condition monitoring of REBs has increasingly played a significant role, especially in high-value and high-impact machines [5,6].

Rolling element bearings can fail in various ways, often affecting specific components. Common failure modes include inner race (IR) failure, outer race (OR) failure, rolling element or ball failure, and cage failure. IR failure occurs due to fatigue cracks initiating at the inner raceway surface, leading to spalling and eventual fracture. Excessive radial load, misalignment, and inadequate lubrication are common causes. OR failure is similar, with fatigue cracks developing on the outer raceway surface. Overloading, misalignment, and contamination are primary causes. Ball failure, affecting balls or rollers, can result in surface pitting, spalling, fracture due to excessive load, poor lubrication, and contamination. Finally, the cage, which separates the rolling elements, can experience fatigue, wear, and fracture due to inadequate lubrication, excessive vibration, and misalignment [7].

Early-stage fault detection has traditionally relied on signal processing techniques in the time, frequency, and time-frequency domains. However, since 2010, machine learning (ML)-based methods have gained popularity for improving diagnostic accuracy [8,9]. Initially, traditional machine learning (ML) methods, such as statistical approaches and basic classification techniques, relied on manually extracted features combined with linear classification models [10]. While these methods offered modest improvements in diagnostic accuracy, their capabilities remained limited. Nevertheless, they laid the groundwork for more advanced techniques. Traditional ML approaches have encountered two major challenges. First, they require extensive data for model training, significant computational resources, a complex feature extraction process, and substantial domain expertise. Second, they depend on training and testing data from identical machines operating under similar conditions, which restricts their generalizability and limits their applicability in real-world industrial settings [11].

Traditionally, vibration-based bearing fault detection has relied on classical signal processing techniques for feature extraction. One of the most widely used methods is the high-frequency resonance technique (HFRT), which detects bearing characteristic frequencies (BCFs), such as BPFO, BPFI, BSF, and FTF [12]. While HFRT effectively enhances fault-related features, it requires the careful selection of a band-pass filter [13]. This process is challenging without human expertise, as resonance frequencies vary depending on machine type and operating conditions. Moreover, HFRT’s performance can be affected by background noise and unrelated frequency components, potentially compromising fault detection accuracy [14]. To overcome these limitations, this study introduces a novel hybrid approach [15] that automates HFRT by integrating cepstrum pre-whitening (CPW), high-pass filtering, moving quartile averaging, and amplitude normalization. These enhancements help suppress background noise, remove irrelevant frequency components, and mitigate domain shifts between simulated and real-world vibration signals, ultimately improving fault detection accuracy.

In recent years, deep learning (DL) has emerged as a transformative approach, introducing neural network architectures capable of automatically learning complex feature representations from raw data. Models such as convolutional neural networks (CNNs) have significantly improved fault classification accuracy in bearing monitoring by directly processing raw vibration signals and capturing intricate patterns that traditional methods might miss.

Despite their enhanced pattern recognition capabilities, DL-based models still face critical challenges. Like classic ML methods, they struggle with data availability, particularly in obtaining labelled and structured data. Additionally, they face challenges in data distribution for generalized model development. Current DL models typically assume that training and testing data share similar distributions [16]. However, this assumption often does not hold when transitioning from laboratory settings to real-world applications, which involve a much wider range of bearing designs and operating conditions. These variations can lead to significant discrepancies, reducing diagnostic accuracy and limiting the practical application of ML models in industrial bearing condition monitoring [17]. The effectiveness of DL models heavily depends on large volumes of labelled data for training. However, collecting such data is labour-intensive, time-consuming, and impractical in real machines. This is especially true because machines do not intentionally operate under faulty conditions due to the risk of severe equipment damage and safety hazards for operators [18].

In recent years, the introduction of transfer learning (TL) techniques has marked a significant milestone in intelligent bearing fault diagnosis models [18]. TL addresses challenges related to data availability and domain adaptation by transferring knowledge from one domain to another to enhance model performance. When effective, TL-based models can monitor machines with similar configurations under different operating conditions to a certain extent [19], providing some degree of generalizability. Although TL-based approaches have significantly advanced bearing condition monitoring, their real-world implementation remains limited due to the complexities of industrial machines. Model performance tends to drop significantly when tested on entirely new data sources, such as machines with different specifications or operating environments. Additionally, TL methods require access to a target-domain dataset during training—whether labelled or unlabelled—to analyse distribution discrepancies. However, in industrial settings, obtaining a comprehensive target-domain dataset that includes all health states is particularly challenging, further complicating the deployment of TL-based models [20].

In 2019, Yang et al. [20] proposed a feature-based transfer neural network (FTNN) that leveraged transfer learning to diagnose bearing health by transferring knowledge between different bearings. A CNN was used to extract transferable features from vibration data, incorporating domain adaptation and pseudo-labelling techniques. The model was validated on three transfer tasks (Tasks A–D, B–D, and C–D), where datasets A, B, and C served as source domains and dataset D as the target domain. It achieved an average classification accuracy of 84.32%, outperforming six existing methods. However, when dataset C was used as the source domain, accuracy dropped to 76%. This study emphasized the critical role of unlabelled data quantity and health state diversity in effective pseudo-labelling. Guo et al. [21] introduced a deep convolutional transfer learning network (DCTLN) for diagnosing rolling element bearing (REB) faults using unlabelled target data. Their model integrated a 16-layer 1D CNN for feature extraction and health classification with a domain adaptation module based on maximum mean discrepancy (MMD) to minimize domain discrepancies. Tested on six transfer tasks across datasets from CWRU, IMS, and locomotive bearings, DCTLN achieved classification accuracies exceeding 82%, with some surpassing 89%. The study underscored the importance of diverse datasets and comprehensive test class coverage for reliable model evaluation and practical industrial application.

Bearing vibration data simulation has emerged as a valuable solution for addressing data availability challenges, particularly in the development of bearing fault detection models. Numerical models are used to generate vibration data based on a physical bearing model, which has been instrumental in training fault diagnosis systems [20]. This approach, which relies on simulated data, is especially useful in scenarios where acquiring sufficient real-world data is difficult, such as in bearing fault classification. Simulated data provide a controlled environment to create diverse fault scenarios, allowing models to learn robust fault representations. Studies, such as [22], emphasize the effectiveness of this method, as simulated vibration signals can closely replicate real-world machine dynamics under various operating conditions, making them well-suited for bearing fault diagnosis.

This study aims to develop a generalized machine learning (ML) model capable of detecting bearing faults that were not included in the training process by utilizing simulated vibration data for training a CNN. The trained model will be tested and evaluated using both experimental and real-world test data.

CNNs are chosen for their parameter efficiency, automatic feature extraction, and hierarchical representation learning capabilities, which have been shown to enhance accuracy and robustness in bearing fault classification [23]. The simulated data, generated based on a diverse range of bearing designs defined by manufacturer specifications [24], are further refined by incorporating controlled noise under various operating conditions. Once successfully trained, the CNN model is validated using vibration data from experimental setups and real-world applications to assess its effectiveness.

This paper is organized in the following sections. Section 2 outlines the methodology, encompassing an overview of the model architecture, the simulation of bearing vibration signals, and the CNN algorithm employed. Section 3 delves into the training and testing outcomes. Finally, Section 4 presents the conclusions drawn from this research.

## 2. Methodology

**Figure 1:**
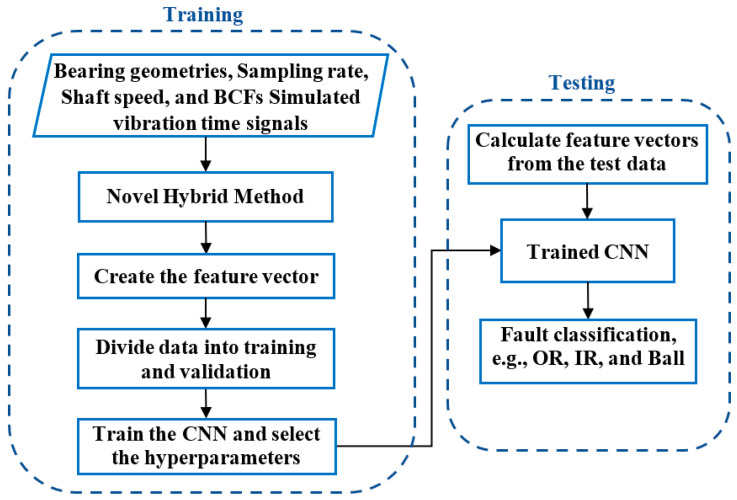
Diagram illustrating the bearing fault classification model using simulated bearing vibration data, the novel hybrid method, and a CNN algorithm.

Figure 1 illustrates a flow-chart outlining the methods used in this study to develop a generalised ML model for bearing fault diagnosis. Model development begins with extracting bearing geometry information from manufacturers’ catalogues [24], considering a wide range of bearings designs. These specifications are then used to generate bearing vibration signals using numerical models developed by [15]. From the simulated vibration signals, 33 values are extracted as input features for ML model development, including BCF harmonics and their corresponding sidebands, as defined in [25]. These features have been shown to capture essential characteristics associated with typical bearing faults [19]. The root mean square (RMS) values of background noise in the signal is derived and used as the 34th feature, representing signal characteristics without BCFs. These 34-feature vectors serve as inputs for CNN model development. This study investigates three types of bearing faults: OR faults, IR faults, and ball faults. Simulated data are used to train and validate CNN models, and the optimised CNN model is then tested using vibration signals collected from various laboratory experiments and engine tests. Fine-tuning may also be performed to enhance model performance, ensuring high accuracy and robust generalization for bearing fault diagnosis [26].

### 2.1. Bearing Vibration Signal Simulation

Due to the challenges of obtaining high-quality vibration signals from experimental testing and real-world machines—especially across a broad range of bearing designs and operating conditions—this study utilizes numerically simulated vibration data to address these limitations.

First, based on the bearing manufacturer’s catalogue, 137 bearing designs are considered, with IR diameters ranging from 10 mm to 150 mm and OR diameters from 19 mm to 320 mm, covering a wide range of applications [24]. Simulated vibration signals with bearing faults on the OR, IR, and ball or roller elements are generated using the formulas outlined in [15]. To enhance realism, various signal-to-noise ratio (SNR) values, shaft speeds, and sampling rates are randomly selected within a predefined range, as shown in Table 1. These values are based on observations from experimental and real-world datasets within the field [27,28,29], including data from electric motors, dynamometers, civil jet engines, and subscale jet engine test rigs.

**Figure 2:**
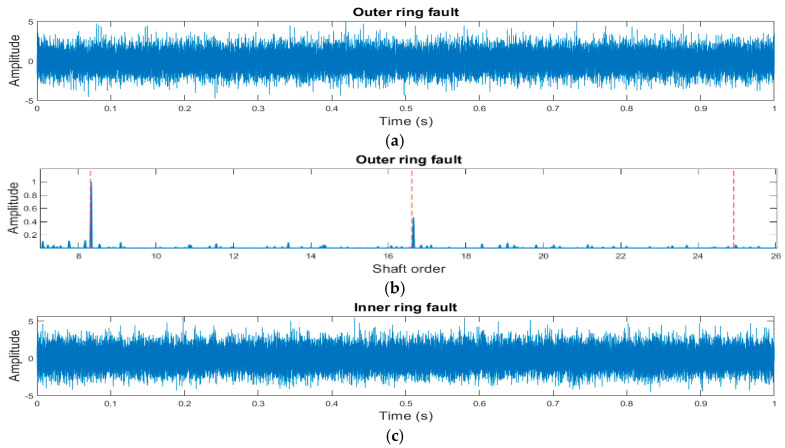

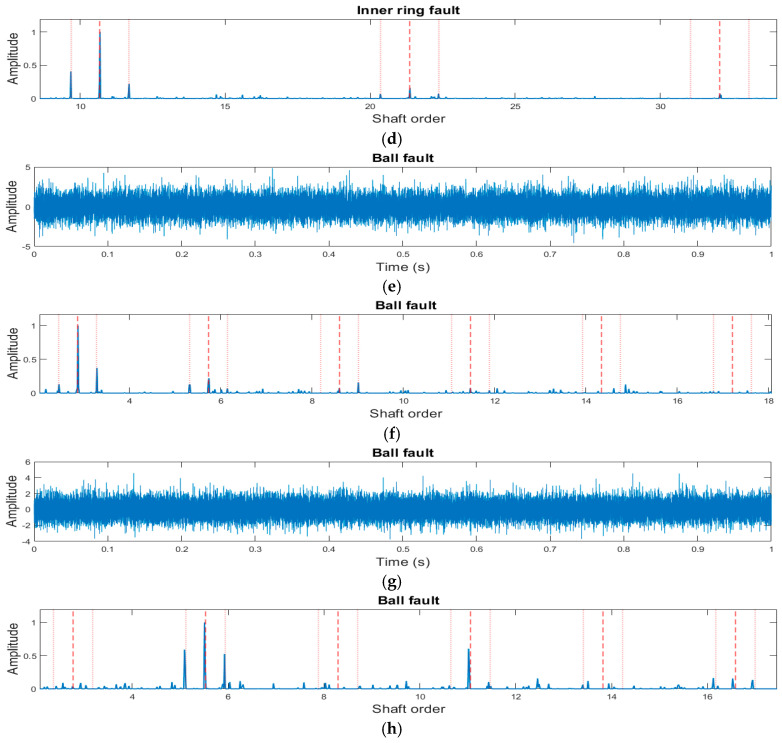
Simulated vibration signals and corresponding spectra generated using the novel hybrid method. (**a**) Simulated time signal depicting a bearing with an OR fault. (**b**) Spectrum obtained using the novel hybrid method for the OR fault. (**c**) Simulated time signal representing a bearing with an IR fault. (**d**) Spectrum obtained using the novel hybrid method for the IR fault. (**e**) Simulated time signal of a bearing with a ball fault where the fault contacts one of the races. (**f**) Spectrum derived using the novel hybrid method for the ball fault contacts one of the races. (**g**) Simulated time signal of a bearing with a ball fault where the fault contacts both races. (**h**) Spectrum obtained using the novel hybrid method for the ball fault contacts both races.

A total of 1200 one-second data samples have been generated, with 400 samples for each of the three bearing fault types. Figure 2, presents examples of these time-series signals alongside their corresponding frequency domain plots after being processed using the novel hybrid method developed by the authors [15]. The figure highlights relevant faults in both the time domain (impulses) and the frequency domain (BCF components).

For ball faults, two scenarios are considered: one where the fault contacts both the IR and OR and another where it contacts only one of the races [30]. Based on the frequency spectra, a feature vector of 34 parameters is constructed for each signal. This includes 33 BCF-related features and the root mean square (RMS) value of background noise, calculated from the spectrum after removing the BCF components to isolate noise characteristics.

### 2.2. Convolutional Neural Network (CNN) Algorithm

**Figure 3:**
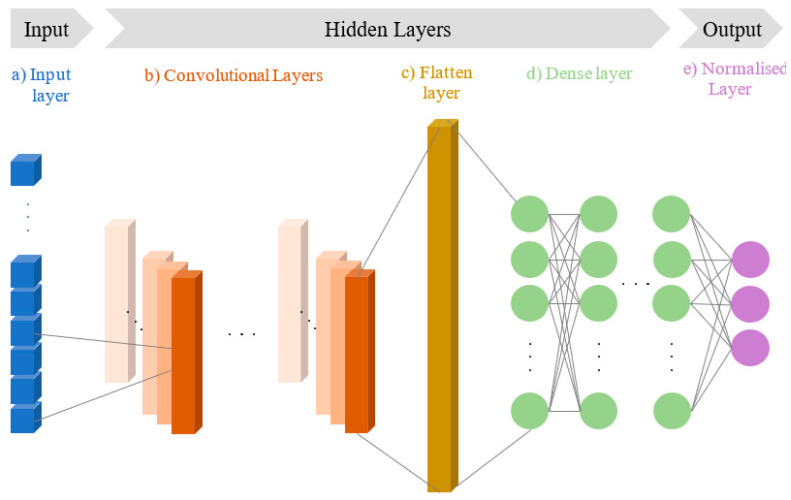
An illustration of a typical 1D-CNN architecture.

With the defined input feature vector of 34 elements, this study employs a one-dimensional convolutional neural network (1D-CNN) architecture. 1D-CNNs are a specialized variant of standard CNNs designed for processing sequential data, such as one-dimensional vectors and time-series signals. While they follow a similar structure to 2D-CNNs, their convolution operations are performed along a single spatial dimension [31]. As illustrated in Figure 3, the convolutional layers utilize a set of 1D convolutional kernels that slide across the input feature vector to generate feature maps. The flatten layer then converts the multi-dimensional output of these convolutional layers into a one-dimensional vector, making it suitable for processing by the dense layers. The dense layer, which may consist of multiple hidden layers, acts as a classifier, learning non-linear feature representations to map the extracted features to the desired output in the final normalized layer.

During training, the kernel weights in the convolutional layers and the weights in the dense layers are optimized to detect and activate specific local patterns in the input data, improving the model’s ability to classify bearing faults accurately.

For a 34-element input vector, an appropriate 1D kernel size ranges from 2 to 5 elements. During training, multiple convolutional layers are stacked, with batch normalization applied after each convolutional layer. Batch normalization [32] helps stabilize and accelerate training by normalizing activations within each mini-batch, allowing for higher learning rates and improving the model’s robustness against poor initialization.

To optimize the 1D-CNN architecture, hyperparameters such as the number of filters per convolutional layer, the number and size of fully connected layers, dropout rates (DRs), and other regularization parameters are fine-tuned throughout the training process.

## 3. Model Development and Test Results

The development of a generalized CNN model involves simultaneously performing training and validation while optimizing hyperparameters using the grid search method [33]. Initially, five convolutional layers and five dense layers are predefined. From these, 25 possible layer combinations are selected for separate training and validation.

Each convolutional layer employs an increasing number of filters. The first layer starts with 3 filters, followed by 6 filters in the second layer. The third layer increases to 12 filters, the fourth to 24 filters, and the fifth incorporates 48 filters. Each of these layers uses a kernel size of 2, enabling detailed feature extraction at progressively deeper levels of the network.

**Figure 4:**
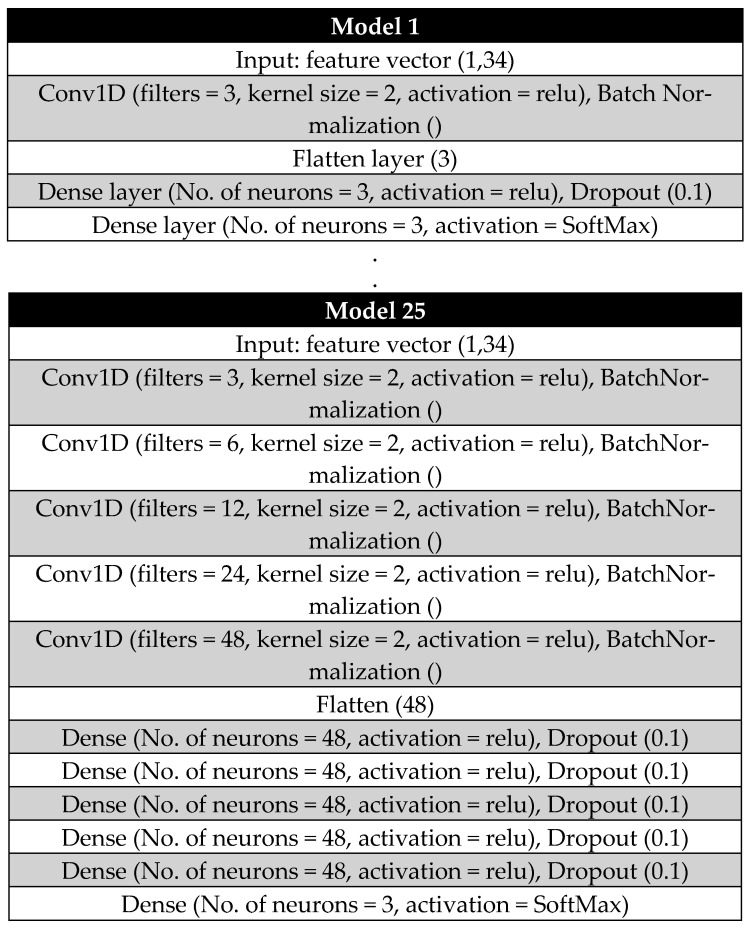
The first and the last predefined CNN models during the random grid optimisation process.

The number of neurons in each dense layer is determined based on the output dimension of the flattening layer following the final convolutional layer. Figure 4 illustrates the architectures of the first and last CNN models used during the random grid optimization process, while Table 2 summarizes the number of convolutional layers (CLs) and dense layers (DLs) for each model.

Throughout the optimization process, key hyperparameters, such as activation functions, learning rate (fixed at 10^−4^), kernel size, and dropout rate (DR, set at 0.1 or 10%), were kept constant to maintain consistency.

To enhance model robustness and prevent overfitting, batch normalization was applied after each convolutional layer, while dropout regularization was used after each dense layer. Additionally, early stopping was implemented using TensorFlow’s *EarlyStopping* callback, which monitored validation loss and halted training if no improvement was observed for 10 consecutive epochs. This approach ensured that the model restored the weights corresponding to the lowest validation loss, optimizing its performance.

### 3.1. Training Results

**Figure 5:**
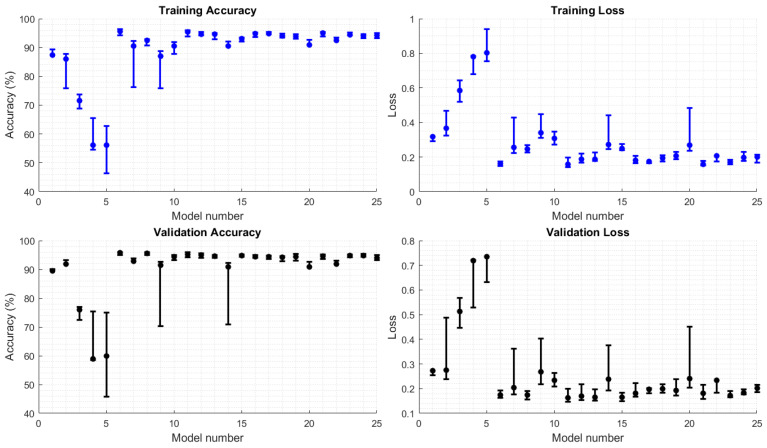
Error bar of training and validation accuracies and loss values for various model architectures during grid search optimization.

To evaluate model accuracy and loss more effectively, each model was trained 10 times with randomly split training and validation data. Specifically, 75% of the simulated samples were allocated for training, while the remaining 25% were used for validation. The performance of each model—including accuracy and loss values—is presented with error bars, where each bar represents the average, the standard deviation below the average, and the standard deviation above the average, based on the 10 training and validation runs. The summarized results are shown in Figure 5.

The first five models, which contain only a single convolutional layer, exhibit lower accuracy and higher loss values. As the number of dense layers increases, accuracy declines further, accompanied by a relatively high variation across different training and validation data clusters. However, introducing a second convolutional layer leads to a significant accuracy improvement and a substantial reduction in loss values for both training and validation datasets.

Among all models, model six demonstrates consistently high accuracy for both training and validation, along with the lowest loss value. Additionally, the short length of its error bars indicates minimal deviation across different data clusters, highlighting its stability and robustness.

**Figure 6:**
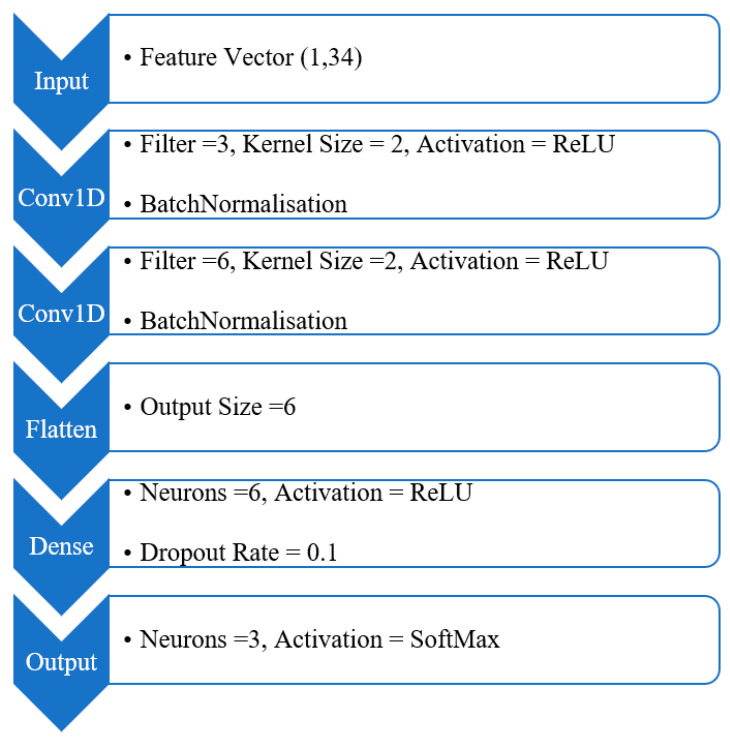
The architecture of the best-performing model (Model 6) based on the grid search optimisation results.

Among the 10 models trained using this architecture, the model with the highest accuracy and lowest loss—referred to as Model 6—was selected for further testing on additional data. Along with its minimal standard deviation in accuracy and loss values, Model 6 demonstrated superior overall performance. As shown in Figure 6 and the corresponding table, its architecture consists of two convolutional layers and one dense hidden layer.

**Figure 7:**
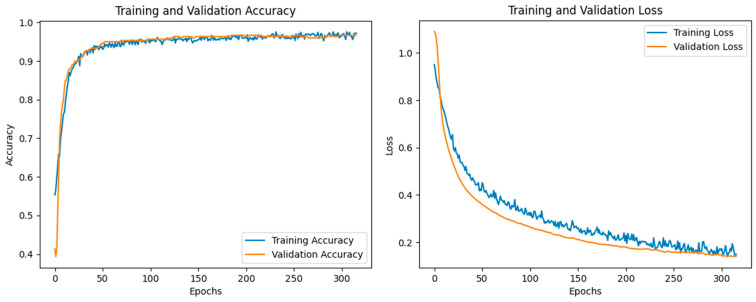
Accuracy and loss values for the training and validation data during the training process of the optimized model (model number 6).

Figure 7 illustrates the accuracy and loss values of the selected optimized model throughout the training process. Based on the *EarlyStopping* callback criteria defined during model development, the training process concluded after 320 epochs. At this point, the model achieved a training accuracy of 97.23% and a validation accuracy of 96.67%. Additionally, the training loss was minimized to 0.15, while the validation loss decreased to 0.14, confirming the effectiveness of the CNN model.

### 3.2. Test Results

To evaluate the generalization ability of the best-performing CNN model (Model 6) trained on simulated vibration data, it was tested on vibration signals collected from three different machines. The two primary test data sources included experimental data from the CWRU database [27] and ground test data from a Safran jet engine [29].

The CWRU dataset consists of vibration data obtained from two different bearing test rigs with seeded faults introduced into bearing components. In contrast, the Safran jet engine dataset includes real-world vibration signals recorded during engine tests conducted under both constant and run-up speed conditions, where faults were observed to develop naturally over time. These diverse test conditions provide a robust evaluation of the model’s ability to generalize beyond simulated training data.

### 3.3. CWRU Bearing Fault Diagnosis Results

The CWRU dataset includes three distinct classes of faulty bearings, with seeded faults introduced in the OR, IR, and ball at varying severity levels. These faults were applied to two separate bearings: the drive-end (DE) bearing and the fan-end (FE) bearing. During data collection, the test rig had to be repeatedly disassembled and reassembled to modify the fault type or severity by replacing faulty components. However, this process inadvertently introduced an additional mechanical fault, which distorted some vibration samples and masked the bearing fault signatures in the collected signals.

In a benchmark study, Randall et al. [34], analysed the dataset using three different bearing fault diagnosis methods and identified mechanical looseness as the likely cause of these distortions. This issue introduced sharp impacts in the vibration signals, evident in the frequency spectrum through the presence of numerous shaft harmonics. These harmonics could attenuate or even obscure the actual bearing fault signatures.

To address this, Randall et al. classified the collected vibration samples into three categories: diagnosable, partially diagnosable, and non-diagnosable. Each category was further divided into two levels, as specified in Table 3. The percentage distribution of these health states within the dataset is presented in Table 4.

**Figure 8:**
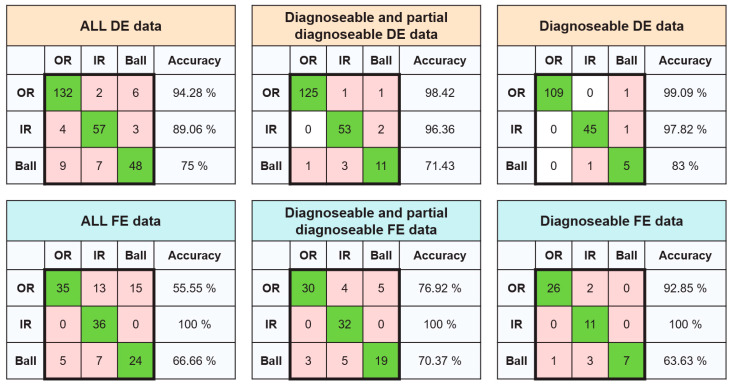
Confusion matrices of test results for the CWRU DE and FE bearings, categorized into diagnosable, partially diagnosable, and non-diagnosable vibration samples.

Following the classification framework proposed by Randall et al. [30], the CNN model developed in this study was tested using different categories of data. The results, shown in Figure 8, provide a comparative analysis. For the DE bearing (Figure 8, top row), when tested on the entire dataset, the model achieved an average classification accuracy of 88.42%, with high classification rates for OR faults (94.28%) and IR faults (89.06%). However, the model struggled to classify ball faults, achieving only 75% accuracy.

When the dataset was refined to include only “diagnosable and partially diagnosable” samples, the model’s performance significantly improved. The classification accuracy increased to 98.42% for OR faults and 96.36% for IR faults. However, the accuracy for ball fault classification dropped slightly to 71.43%, likely due to the small sample size (only 15 samples in this category). Further narrowing the dataset to include only “diagnosable” samples resulted in the highest classification performance. The model achieved 99.09% accuracy for OR faults, 97.82% for IR faults, and 83% for ball faults. The relatively lower accuracy for ball faults can be attributed to poor data quality, as indicated in Table 4.

For the FE bearing (Figure 8, bottom row), the model exhibited similar trends to those observed for the DE bearing across the three data categories. However, its overall performance was notably lower, particularly for OR and ball faults.

One key exception was the model’s classification accuracy for IR faults, where it achieved 100% accuracy across all three categories. This result highlights the model’s generalizability and robustness to noise and variations in data quality.

Overall, the trend in results aligns with the data quality observations in Table 4, yet with significant improvements. When compared to the classifications by Randall et al. [34] in Table 4, the CNN model in this study demonstrates a strong ability to detect OR and IR faults. Specifically, it correctly diagnosed 94.28% of OR faults in the DE bearing and 55.55% of OR faults in the FE bearing when using all data, outperforming Randall et al.’s reported detection rates of 78.5% and 46.6%, respectively.

Guo et al. [21] developed a transfer learning model using vibration samples from the CWRU dataset, specifically from a 2 HP motor operating at a shaft speed of 1750 rpm. Their model, which emphasized domain adaptation and distribution discrepancy metrics during fine-tuning, achieved an average accuracy of 86.6%. Additionally, when tested on the DE bearing using the full dataset, their model attained an average classification accuracy of 88.42%. In contrast, the CNN model developed in this study achieved significantly higher bearing fault classification accuracies, despite not using CWRU data for training. This demonstrates the model’s superior generalization ability and effectiveness in classifying faults across different datasets.

### 3.4. Safran Jet Engine Bearing Fault Diagnosis Results

**Figure 9:**
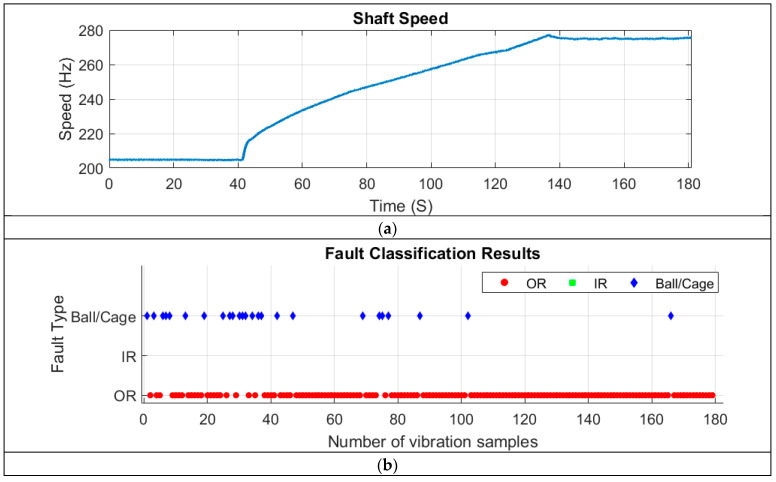
(**a**) Shaft speed profile during the ground test and (**b**) fault classification results obtained using the trained CNN for the Safran jet engine ground test.

To further assess the generalisability of the CNN model developed in this study, vibration data collected from an accessory gearbox of a Safran jet engine during a ground test were used as input. Vibration and tachometer signals were continuously recorded for 180 s as the engine gradually accelerated from idle to full power (see speed profile in Figure 9a). During the test, two faults were reported: an outer ring (OR) fault and a cage fault in the rolling element bearing (REB) located on a shaft within the accessory gearbox [29].

To diagnose bearing faults in the vibration signals using the CNN model developed in this study, the 180-s continuous vibration signal was segmented into 180 one-second samples. The diagnosis results are presented in Figure 9b, where the model clearly detected both outer ring (OR) faults and ball/cage faults.

During the first 50 s, when the shaft speed was constant and low, the CNN model successfully identified both faults. Throughout the speed run-up period (approximately 40 to 140 s), the model predominantly detected OR faults, along with a few segments showing ball/cage faults. In the high-speed phase (after 140 s), nearly all signals indicated an OR fault, with only one segment (around 165 s) showing a ball/cage fault.

Overall, the CNN model accurately classified 86.03% of the samples as OR faults, aligning with the data labels provided by Safran [29]. Additionally, 13.97% of the samples were classified as ball/cage faults, primarily occurring during the ground test.

This highlights that as shaft speed increases, OR fault-related features exhibit a higher signal-to-noise ratio (SNR) compared to cage fault features. As a result, the CNN model—designed for single-fault diagnosis—primarily detected the OR fault during the run-up and high-speed conditions.

These findings clearly demonstrate the model’s ability to detect multiple faults under noisy and transient operating conditions, without requiring additional training. Notably, the model’s ability to identify faults during the speed run-up stage is particularly significant for real-world applications, where early fault detection can enhance maintenance strategies and prevent severe machine failures.

## 4. Conclusions

This study has developed a CNN-based bearing fault diagnosis model trained on numerically simulated vibration data covering a wide range of bearing designs, demonstrating high generalizability. The model leverages input features based on bearing characteristic frequencies (BCFs), identified using a novel hybrid method developed by the authors, to reduce domain shifts and automate feature extraction.

By generating a large volume of bearing vibration data with diverse fault scenarios using well-established numerical methods, the model effectively overcomes key challenges in ML-based learning, particularly data scarcity and domain adaptation.

Following extensive training, which involved tuning numerous hyperparameters, an optimized 1D CNN model with two convolutional layers and one dense layer (see Figure 6) was developed for bearing fault diagnosis. The model’s generalizability was rigorously tested using real-world data from both laboratory experiments and a jet engine.

The results demonstrate an average accuracy of 95.73% for diagnosable DE and FE vibration samples from the CWRU dataset, significantly outperforming the 86.6% accuracy reported in benchmark studies using transfer learning (TL)-based models.

Also, compared to our previous work [25] with classical ML models like SVM and logistic regression, the CNN-based approach achieved better accuracy and robustness in bearing fault diagnosis.

When applied to the Safran jet engine test data, the model accurately identified existing faults. Although it was designed for single-fault diagnosis, it successfully detected ball/cage faults in 13.97% of samples, demonstrating robust performance in high-noise, non-stationary operating conditions and reinforcing its reliability for real-world applications.

This study makes a significant contribution to the field of intelligent bearing fault classification by introducing a generalized model that effectively addresses training data scarcity in real-world scenarios. Future work will focus on enhancing the model’s capabilities to achieve a generalised classification of both healthy and faulty bearing states across diverse operational conditions and various machine types.

## Figures and Tables

**Table 1:** Ranges of SNR values, shaft speed, and frequency sampling rate used for the signal simulation to provide the training data for the CNN model.

Time-Domain SNR	Shaft Speed (Hz)	Sampling Rate (kHz)
[−5,−10]	[25,50]	[20,50]

**Table 2:** Summary of the number of convolutional layers (CLs) and dense layers (DLs) for each CNN model during the grid search.

Model Number	CLs	DLs	Model Number	CLs	DLs	Model Number	CLs	DLs
1	1	1	10	2	5	19	4	4
2	1	2	11	3	1	20	4	5
3	1	3	12	3	2	21	5	1
4	1	4	13	3	3	22	5	2
5	1	5	14	3	4	23	5	3
6	2	1	15	3	5	24	5	4
7	2	2	16	4	1	25	5	5
8	2	3	17	4	2			
9	2	4	18	4	3			

**Table 3:** CWRU data categorisation of diagnosis outcomes based on the literature results [34].

Diagnosis Category	Diagnosis Success	Explanation
Y1	Yes	Data clearly diagnosable and showing classic characteristics for the given bearing fault in both the time and frequency domains.
Y2	Yes	Data clearly diagnosable but showing non-classic characteristics in either or both of the time and frequency domains.
P1	Partial	Data probably diagnosable, e.g., envelope spectrum shows discrete components at the expected fault frequencies but they are not dominant in the spectrum.
P2	Partial	Data potentially diagnosable, e.g., envelope spectrum shows smeared components that appear to coincide with the expected fault frequencies.
N1	No	Data not diagnosable for the specified bearing fault but with other identifiable problems (e.g., looseness).
N2	No	Data not diagnosable and virtually indistinguishable from noise, with the possible exception of shaft harmonics in the envelope spectrum.

**Table 4:** Percentage of diagnosable, partial diagnosable, and non-diagnosable vibration samples in CWRU dataset [25].

Bearing	Health State	Diagnosable (%)	Partial Diagnosable (%)	Non-Diagnosable
Drive end	OR fault	78.5	12.2	9.3
	IR fault	73.4	14.1	12.5
	Ball fault	10.9	10.9	78.2
Fan end	OR fault	46.6	18.33	35
	IR fault	52.78	36.11	11.11
	Ball fault	30.5	44.5	25

## Data Availability

The experimental data used in this study, are available in public domain of the literature.
